# Induction of pulmonary HLA-G expression by SARS-CoV-2 infection

**DOI:** 10.1007/s00018-022-04592-9

**Published:** 2022-11-05

**Authors:** Barbara Seliger, Simon Jasinski-Bergner, Chiara Massa, Anja Mueller, Katharina Biehl, Bo Yang, Michael Bachmann, Danny Jonigk, Philip Eichhorn, Arndt Hartmann, Claudia Wickenhauser, Marcus Bauer

**Affiliations:** 1 Institute of Medical Immunology, Martin Luther University Halle-Wittenberg, Magdeburger Str. 2, 06112 Halle (Saale), Germany; 2 Fraunhofer Institute for Cell Therapy and Immunology, 04103 Leipzig, Germany; 3 Institute of Translational Immunology, Medical School “Theodor Fontane”, 14770 Brandenburg, Germany; 4 Helmholtz Zentrum Dresden-Rossendorf (HZDR), Institute of Radiopharmaceutical Cancer Research, Dresden, Germany; 5 Institute of Pathology, Hannover Medical School, 30625 Hannover, Germany; 6 German Center for Lung Research (DZL), Hannover Medical School (BREATH), 30625 Hannover, Germany; 7 Institute of Pathology, Friedrich-Alexander University, 91054 Erlangen, Germany; 8 Institute of Pathology, Martin Luther University Halle-Wittenberg, 06112 Halle (Saale), Germany

**Keywords:** HLA-G, SARS-CoV-2, microRNA, Immune cell infiltration, Immune response

## Abstract

The non-classical human leukocyte antigen (HLA)-G exerts immune-suppressive properties modulating both NK and T cell responses. While it is physiologically expressed at the maternal–fetal interface and in immune-privileged organs, HLA-G expression is found in tumors and in virus-infected cells. So far, there exists little information about the role of HLA-G and its interplay with immune cells in biopsies, surgical specimen or autopsy tissues of lung, kidney and/or heart muscle from SARS-CoV-2-infected patients compared to control tissues. Heterogeneous, but higher HLA-G protein expression levels were detected in lung alveolar epithelial cells of SARS-CoV-2-infected patients compared to lung epithelial cells from influenza-infected patients, but not in other organs or lung epithelia from non-viral-infected patients, which was not accompanied by high levels of SARS-CoV-2 nucleocapsid antigen and spike protein, but inversely correlated to the HLA-G-specific miRNA expression. High HLA-G expression levels not only in SARS-CoV-2-, but also in influenza-infected lung tissues were associated with a high frequency of tissue-infiltrating immune cells, but low numbers of CD8^+^ cells and an altered expression of hyperactivation and exhaustion markers in the lung epithelia combined with changes in the spatial distribution of macrophages and T cells. Thus, our data provide evidence for an involvement of HLA-G and HLA-G-specific miRNAs in immune escape and as suitable therapeutic targets for the treatment of SARS-CoV-2 infections.

## Introduction

The human leukocyte antigen (HLA)-G is a non-classical HLA class I molecule, which was first characterized by its expression at the feto-maternal interface. In contrast to classical HLA class I antigens, HLA-G is less polymorphic and exists in seven different isoforms, from which four are membrane bound (HLA-G1 to -G4) and three are soluble forms (sHLA-G5 to -G7) [1]. In addition, some novel HLA-G isoforms have been recently identified, but their functions have not yet been determined [2, 3]. In addition to alternative splicing, sHLA-G1 can be generated by proteolytic cleavage mainly mediated by the activity of matrix metalloproteinases (MMPs) [4]. Under physiologic conditions, HLA-G has a highly restricted tissue distribution and is mainly expressed on immune-privileged organs, i.e., thymus, cornea, testis, erythroblasts, mesenchymal stem cells, and cytotrophoblasts. In these tissues, the most common HLA-G isoforms are HLA-G1, sHLA-G1 and HLA-G5 [5]. HLA-G exerts immune-suppressive properties on CD8^+^ cytotoxic T lymphocytes (CTL), natural killer (NK) cells, B cells and dendritic cells by interacting with the appropriate immune cell inhibitor receptors, in particular the Ig-like transcript (ILT)-2, ILT-4, the killer cell immunoglobulin-like receptor (KIR)-2DL4, NKG2A/CD94 and CD160 [5–9]. Next to the interactions with its receptors, the HLA-G-associated tolerance induction could be also mediated by intracellular transfer mechanisms, such as trogocytosis, extracellular vesicles (EV), or tunneling nanotubes [10–12].

The expression of HLA-G is tightly regulated at distinct levels [13]. These include a transcriptional up-regulation of HLA-G by various cytokines, such as interferon (IFN)-ƴ and tumor necrosis factor (TNF)-α, and growth factors, like, e.g., the transforming growth factor (TGF)-β, granulocyte–macrophage colony stimulating factor (GM-CSF), and granulocyte colony stimulating factor (G-CSF) [14–16] as well as microenvironmental factors including indoleamine 2,3-dioxygenase (IDO), hypoxia, metabolites and stress factors, such as heat shock and chemicals, [13, 17]. In addition, HLA-G is altered by epigenetic processes mediated by methylation and histone acetylation [18] or by post-transcriptional control [19]. The latter is due to RNA-binding proteins (RBPs) and/or microRNAs (miRNAs) directed against the 3ʹ untranslated region (UTR) or the coding sequence (CDS) of HLA-G, thereby inhibiting the HLA-G expression [20–24]. In addition, the long non-coding RNA HOX transcript anti-sense RNA (HOTAIR) has been shown to downregulate HLA-G expression [25].

During the last two decades, highly variable levels of HLA-G neo-expression leading not only to escape from immune surveillance, but also to tolerogenic responses of transplants have been described in different neoplasms, autoimmune, and inflammatory diseases as well as upon pathogen infection by parasites, bacteria, and viruses [26–35]. In addition, the pathophysiologic neo-expression of HLA-G surface antigens and of sHLA-G isoforms in tumors or upon viral infections was associated with disease progression, poor clinical outcome, and adverse therapy response of patients [36–46]. Recently, evidence accumulated that HLA-G is an emerging susceptibility and/or protection relevant factor for unresolved virus infection and viral resistance [47]. Thus, targeting of HLA-G, its receptors, or HLA-G-relevant molecules might offer a novel therapeutic strategy for malignant tumors and also viral infections.

The severe acute respiratory syndrome coronavirus-2 (SARS-CoV-2) is a novel RNA beta coronavirus, which causes the coronavirus disease-2019 (COVID-19) [48]. In March 2020, the World Health Organization (WHO) declared COVID-19 as a pandemic public health disease. This disease is associated with infection of the upper respiratory tract with the risk of sustaining a pneumonia and/or an acute respiratory distress syndrome (ARDS) accompanied by a high patients’ morbidity and mortality rate depending on the virus strain [49]. SARS-CoV-2 enters the host cells through its spike protein by binding to the angiotensin-converting enzyme 2 (ACE2) receptor, which is abundantly expressed on alveolar type II epithelial cells of the respiratory tract [50]. SARS-CoV-2 infection often causes an immune deregulation including sustained cytokine production and hyperinflammation, which in turn is associated with disease severity and induces damages to host tissues [51]. Immune profiling of peripheral blood or bronchioalveolar fluid as well as damaged lung tissues has revealed major changes in the immune system of COVID-19 patients [52, 53]. A unique immunological profile was found in the peripheral blood of COVID-19 patients with an increased number of NK cells, but low T cell numbers and overexpression of T cell immunoglobulin and mucin domain (TIM)-3,  programmed cell death ligand 1 (PD-L1) and CD69 in both immune effector cells suggesting a hyperactivated and exhausted immune response upon COVID-1 infection [54]. However, an increased understanding of the immunogenicity in combination with immune responses is urgently needed and will provide further information about the pathophysiologic role of SARS-CoV-2 and the clinical manifestation of severe disease [55, 56]. Regarding a putative link between HLA-G and COVID-19 infection, it has been suggested that in an early inflammatory stage, the host might produce the anti-inflammatory cytokine IL-10, which could later enhance HLA-G expression to avoid injuries [57]. These data were further extended by a positive correlation next to IL-10 with IL-6 and IL-8 in the acute phase of the SARS-CoV-2 infection [58]. Thus, HLA-G expression and/or secretion might reflect a negative feedback response to inflammatory processes during viral infections [29]. Despite HLA-G has been suggested to have immune regulatory functions in SARS-CoV-2-infected patients [59–61], a thorough analysis of HLA-G in COVID-19 patients is still lacking. To elucidate the role of HLA-G during COVID-19, membraneous HLA-G expression in organs of SARS-CoV-2-infected patients and respective controls, its spatial context, and interplay with immune cells were determined and correlated with clinical parameters.

## Materials and methods

### Patients’ characteristics

Formalin-fixed, paraffin-embedded (FFPE) lung tissue samples from 65 SARS-CoV-2-affected patients were collected in Germany between 2020 and 2021 at the Institutes of Pathology of the University Hospital of the Martin-Luther University Halle-Wittenberg, the University Hospital of the Friedrich-Alexander University Erlangen-Nuremberg and of the Hannover Medical School, Hannover. Tissue samples from lungs of patients who died from influenza (*n* = 12) or from heart attack (*n* = 10) were collected in the period from 2009 to 2020 and served as controls. In a subset of SARS-CoV-2-infected patients, FFPE tissues from further organs were available (see Table 1). The FFPE tissues derived from autopsies, or in the case of six patients that survived COVID-19, were obtained by interventional tissue resection during the disease. Clinical and laboratory data were collected from the medical records. SARS-CoV-2 and influenza infections were proven by real-time polymerase chain reaction (RT-PCR) during the life-time of the patients. All samples analyzed are summarized in Table 1.

**Table 1:** Clinico-pathological characteristics and sample specifications

Category	SARS-CoV-2	Influenza	Control
Number of patients	65	10	12
Clinical data			
Age min–max (mean)	36–96 (71)	34–84 (64)	54–83 (69)
Gender			
Male	43	6	7
Female	22	4	5
Survival time			
< 7 days	17		
> 7 days	42		
Survived	6		
Tissues analyzed for HLA-G expression			
Lung	65	10	12
Brain	10	–	–
Heart	12	–	–
Kidney	21	–	–
Pancreas	12	–	–
Spleen	6	–	–
Liver	6	–	–
Samples analyzed for miRNA expression	20	5	5
Samples analyzed by MSI	26	8	5
Histological pattern of acute interstitial pneumonia			
Exudative pattern	12	–	–
Organizing pattern	52	–	–
Fibrosis	1	–	–

Cohorts were stratified based on COVID-19 death data from the Robert Koch Institute as follows: 1st wave comprises calendar weeks 10–31 in 2020. Calendar weeks 32 in 2020 to 11 in 2021 were selected for the 2nd wave [8]. The 3rd wave was defined as beginning of calendar weeks 12–32 in 2021, and the 4th wave as calendar weeks 33–53 in 2021.

### Ethical approval

Autopsies were in accordance with the ethical standards of the institutional and/or national research committee and with the 1964 Helsinki Declaration and its later amendments or comparable ethical standards. All autopsies were conducted after consent for autopsy was obtained from the deceased or next of kin, or autopsies were requested by the health authorities or by the prosecutor’s office. Each participating center had local ethical approval. Clinical parameters were recovered from the final biopsy/autopsy report or the laboratory information system. Only cases with a positive SARS-CoV-2 test (usually antigen tests from either nasopharyngeal or PCR from either nasopharyngeal swab or tissue) either preclinical, clinical, or post-mortem, were implemented in the analyses. Regarding the cause of death, data were taken from the final autopsy report.

The use of collected FFPE tissue samples and serum/plasma samples from patients who survived COVID-19 was approved by the Ethical Committees of the Medical Faculty of the Martin Luther University Halle-Wittenberg, Halle (2017-81), of the MHH Hannover (9022-BO.K-22c), and of the Medical Faculty of the University Hospital in Erlangen.

### Standard morphological evaluation

Hematoxylin and eosin (HE)-stained tissue slides were used to evaluate the specific pattern of damage following SARS-CoV-2 infection with respect to the following histological patterns of acute interstitial pneumonia: acute exudative, organized, and fibrotic pattern [62, 63]. Furthermore, tissue-infiltrating lymphocytes in the alveolar walls were quantified in accordance to the evaluation of stromal tissue-infiltrating lymphocytes (TILs) using the guidelines of the International Working Group for tumor-infiltrating lymphocytes [64].

### Immunohistomorphological evaluation

Conventional immunohistochemistry (IHC) was performed on 3 µm thick, consecutive sections of FFPE samples with the Bond Polymer refine detection Kit (Leica, DS9800) according to the manufacturer’s instructions on a fully automated IHC stainer (Leica Bond). For IHC staining, the anti-HLA-G monoclonal antibody (mAb) (Abcam, UK, clone 4H84) was used as recently described [22]. In addition, mAbs directed against CD3 (Labvision, Germany, clone SP7), CD20 (DAKO, California, USA, clone L26), CD56 (Cell marque, Massachusetts, USA, clone MRQ-42) and the SARS-CoV nucleocapsid protein (Rockland Inc., Pennsylvania, USA, clone 200-401-A50) and the spike protein (Abcam, UK, clone ab272504) were employed according to the supplier’s instructions. Sections were examined and imaged with a Zeiss Axiophot microscope (Zeiss, Jena, Germany). Two pathologists (MB and CW), independently and blinded to the clinical data, scored all samples. HLA-G expression was analyzed using a Histoscore as previously described [65]. The relative amount of positively stained cells (%) was multiplied by their intensity from 0 (negative), 1 (weak), 2 (moderate) to 3 (intense) leading to the expression intensity (or H-score) that was further classified as absent (0), low (1–100), intermediate (101–200), or strong (201–300) overall expression.

### Multispectral imaging

Multispectral imaging (MSI) was performed using the basic protocol described in Wickenhauser et al. [66]. Two multiplex panels with different mAbs and opal-dye combinations were used. The first panel included anti-PD-L1 clone E1L3N (Cell Signaling E1L3N, 1:150) in combination with Opal690, anti-Foxp3 clone 236A/E/ (Abcam, 1:00) with Opal540, anti-CD3 clone SP7 (ThermoFisher SP7, 1:100) with Opal570, anti-CD163 clone MRQ-26 (Cell Marque, 1:50) with Opal620 and anti-panCK Ab AE1/AE3 (Dako, 1:150) with Opal520. The second panel comprised anti-TIM-3 (Abcam, ab241332, 1:1000) with Opal 520, anti-PD-1 (Biocare Medical, NAT105, 1:50) with Opal 540, anti-CD8 (DAKO, C8/144b, 1:50) with Opal 570, anti-TIGIT (Biozol, USC-PAN056HU01-1, 1:50) with Opal 620, anti-CD69 (Abcam, ab233396, 1:50) with Opal 650 and anti-HLA-G (Abcam, clone 4H84, 1:100) with Opal 690. After counterstaining with DAPI (Akoya Biosciences, Marlborough, MA), the sections were mounted and scanned with the Vectra Polaris System (Akoya Biosciences, Marlborough, MA) and a mean of 18 regions of interest (ROIs) per slide were taken with a 20 × zoom. The inForm software (Version 2.4.10, Akoya Biosciences) was employed to perform cell segmentation and phenotyping. PhenoptrReports scripts were used within R to evaluate the frequency and density of the different cell types as well as their interspatial relationships.

### RNA extraction, cDNA synthesis, and qPCR analyses

Two 5 µm thick FFPE tissue slides of lung tissues were subjected to the extraction of total RNA using the MasterPure™ Complete DNA & RNA Purification Kit according to the manufacturer’s protocol (Lucigen, Middleton, WI, USA).

For quantification of the selected HLA-G regulatory miRNAs, template-specific cDNA syntheses were performed with the RevertAid First Strand cDNA Synthesis Kit (Thermo Fisher Scientific, Waltham, MA, USA) and miR specific stem-loop primers [67] as published by Jasinski-Bergner et al. [22]. Subsequently, the qPCR analyses were performed by calculation of relative copy numbers. The HLA-G non-relevant and highly abundantly expressed miR-3960 served as a house keeping gene. For the qPCR reactions GoTaq^®^ qPCR Master Mix (Promega, Madison, WI, USA) was employed. The sequences of the stem-loop primers as well as of the respective qPCR primers and their amplification settings are listed in Table 2.

**Table 2:** Summary of the primer sequences and conditions

Primer	Sequence (5ʹ⟶3ʹ)	Application	Condition (°C)
miR-148A SL Rkt	GTCGTATCCAGTGCAGGGTCCGAGGTATTCGCACTGGATACGACACAAAG	cDNA synthesis	42
miR-148A qPCR	GCCCTCAGTGCACTACAGA	qPCR	60
miR-148B SL Rkt	GTCGTATCCAGTGCAGGGTCCGAGGTATTCGCACTGGATACGACACAAAG	cDNA synthesis	42
miR-148B qPCR	GCCCTCAGTGCATCACAGGA	qPCR	60
miR-152 SL Rkt	GTCGTATCCAGTGCAGGGTCCGAGGTATTCGCACTGGATACGACCCAAGT	cDNA synthesis	42
miR-152 qPCR	GCCCTCAGTGCATGACAGA	qPCR	60
miR-3960 SL Rkt	GTCGTATCCAGTGCAGGGTCCGAGGTATTCGCACTGGATACGACCCCCCG	cDNA synthesis	42
miR-3960 qPCR	GCCCGGCGGCGGCGGAGGC	qPCR	60
miR-548q SL Rkt	GTCGTATCCAGTGCAGGGTCCGAGGTATTCGCACTGGATACGACCCGCCA	cDNA synthesis	42
miR-548qfw qPCR	GCCCGCTGGTGCAAAAGTAA	qPCR	60
miR-628-5p SL Rkt	GTCGTATCCAGTGCAGGGTCCGAGGTATTCGCACTGGATACGACCCTCTA	cDNA synthesis	42
miR-628-5p qPCR	GCCCATGCTGACATATTTAC	qPCR	60
miR-744-5p SL Rkt	GTCGTATCCAGTGCAGGGTCCGAGGTATTCGCACTGGATACGACTGCTGT	cDNA synthesis	42
miR-744-5p qPCR	GCCCTGCGGGGCTAGGGCTA	qPCR	60
miR qPCR rev	GTGCAGGGTCCGAGGT	qPCR	60

### Statistics

Statistical analyses were performed employing IBM SPSS statistic packages (version 25) or GraphPad Prism9. Kolmogorov–Smirnov test revealed non-parametric data (*p* < 0.05). The Mann–Whitney *U* test was used to compare clinical data, frequencies of immune cell subpopulations, and immunohistochemical expression pattern. For correlation analysis Pearson’s correlation was performed. *p* values < 0.05 were considered statistically significant. All graphs were created using GraphPad Prism 9.

## Results

### Determination of HLA-G expression in lung tissues from SARS-CoV-2- and influenza-infected patients and histomorphologic normal control lung tissues

**Fig. 1:**
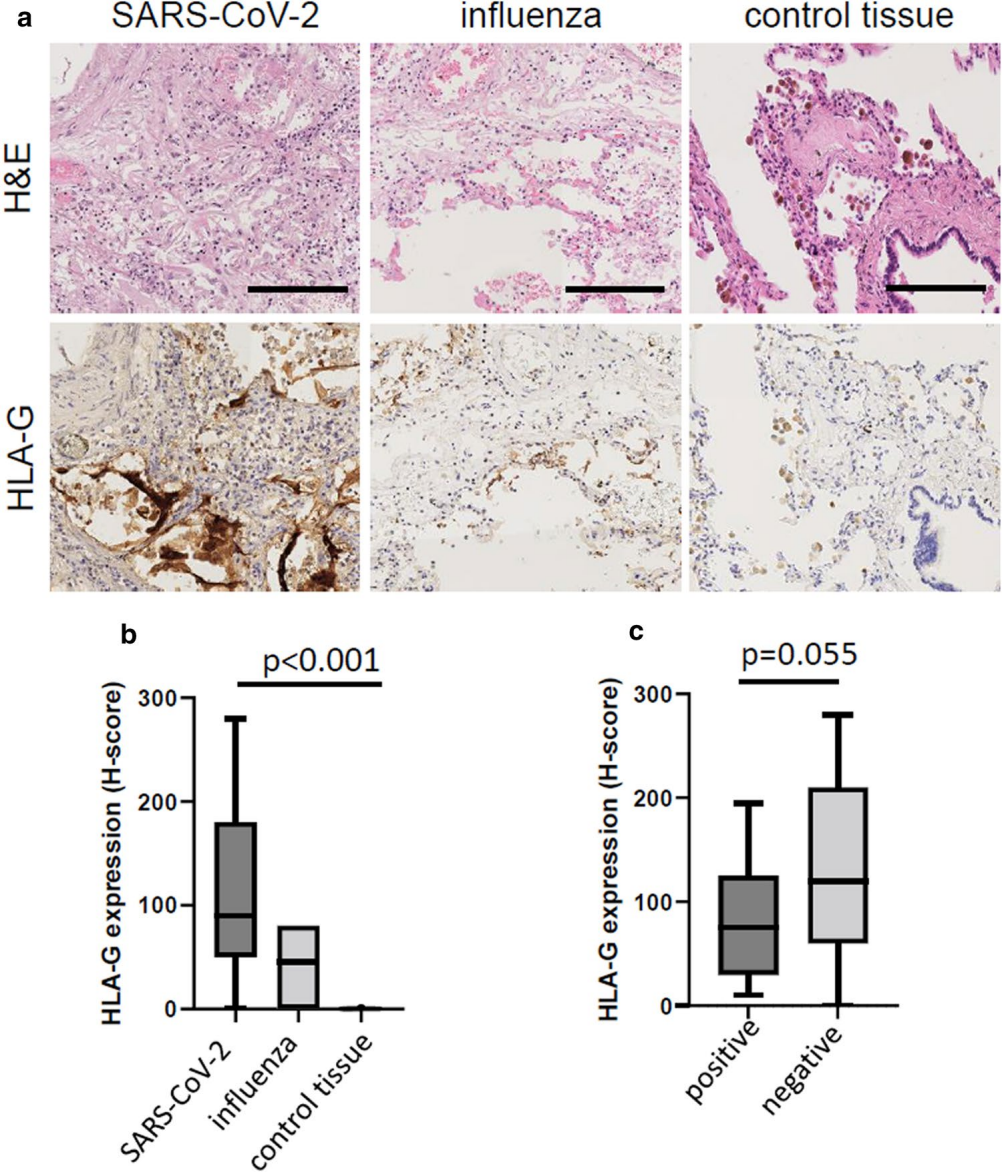
Evaluation of HLA-G expression in lung tissues from SARS-CoV-2- and influenza-infected patients and controls. HLA-G expression in SARS-CoV-2- or influenza-infected lungs as well as in lungs from control patients was determined by IHC as described in “Material and methods”. **a** Representative micrographs of HLA-G expression in lung epithelia from SARS-CoV-2- or influenza-infected patients as well as controls are shown. Magnification 1:40; bars indicate a scale of 50 µm. *H&E *hematoxylin and eosin staining; **b**, **c** HLA-G expression is shown using the H-score for the different patients (SARS-CoV-2, *n* = 65; influenza, *n* = 10; control patients, *n* = 12) or for the SARS-CoV-2-infected individuals divided regarding the presence or absence of detectable nucleocapsid antigen by IHC (positive, *n* = 13; negative, *n* = 52)

To determine whether SARS-CoV-2 infection induces HLA-G expression, lung tissues from SARS-CoV-2- (*n* = 65) or influenza- (*n* = 10) infected patients as well as from respective controls (*n* = 12) were stained by IHC with the HLA-G-specific mAb 4H83 detecting all major HLA-G isoforms. Representative images of the tissue stainings are shown in Fig. 1a demonstrating a highly heterogeneous HLA-G expression pattern in the lung tissues analyzed with a predominant positivity in pneumocytes and weaker expression in immune cells and some cases of bronchial respiratory epithelia. A comparable staining pattern, but statistically significant higher HLA-G expression levels were found in SARS-CoV-2- compared to lung tissues from influenza-infected patients (Fig. 1b). In contrast, pneumocytes of the lung control samples lacked HLA-G expression, while a few HLA-G-positive immune cells were detected (Fig. 1b). To evaluate whether this HLA-G expression was organ specific or also relevant in other organs receptive to SARS-CoV-2-induced damage, HLA-G expression was determined in representative samples of kidney (*n* = 21) and heart tissues (*n* = 12) obtained from SARS-CoV-2-infected patients. All these tissue samples lacked HLA-G expression (data not shown) suggesting a tissue-specific HLA-G neo-expression upon course of COVID-19.

### Correlation of HLA-G expression with SARS-CoV-2 nucleocapsid antigen and spike protein expression

Next to HLA-G, the SARS-CoV-2 nucleocapsid and spike protein antigen expression was determined in the lung tissue samples analyzed using IHC. In the group of SARS-CoV-2-infected patients, the nucleocapsid expression was detectable in 23/65 (35.4%), the spike protein in 31/65 (47.7%) of cases. All samples expressing the nucleocapsid were also positive for the spike protein. The expression of the respective markers was detectable in pneumocytes, respiratory epithelia, and lung infiltrating immune cells, but neither in other tissue samples from SARS-CoV-2-infected patients nor in lung epithelium samples of influenza-infected or control patients. All SARS-CoV-2 nucleocapsid antigen-positive lung samples expressed HLA-G, while the 42 nucleocapsid antigen-negative tissues from patients with proven SARS-CoV-2 infection presented a trend towards higher HLA-G expression levels, although statistically non-significant (Fig. 1c).

### Inverse correlation of HLA-G expression with HLA-G-specific miRNAs

**Fig. 2:**
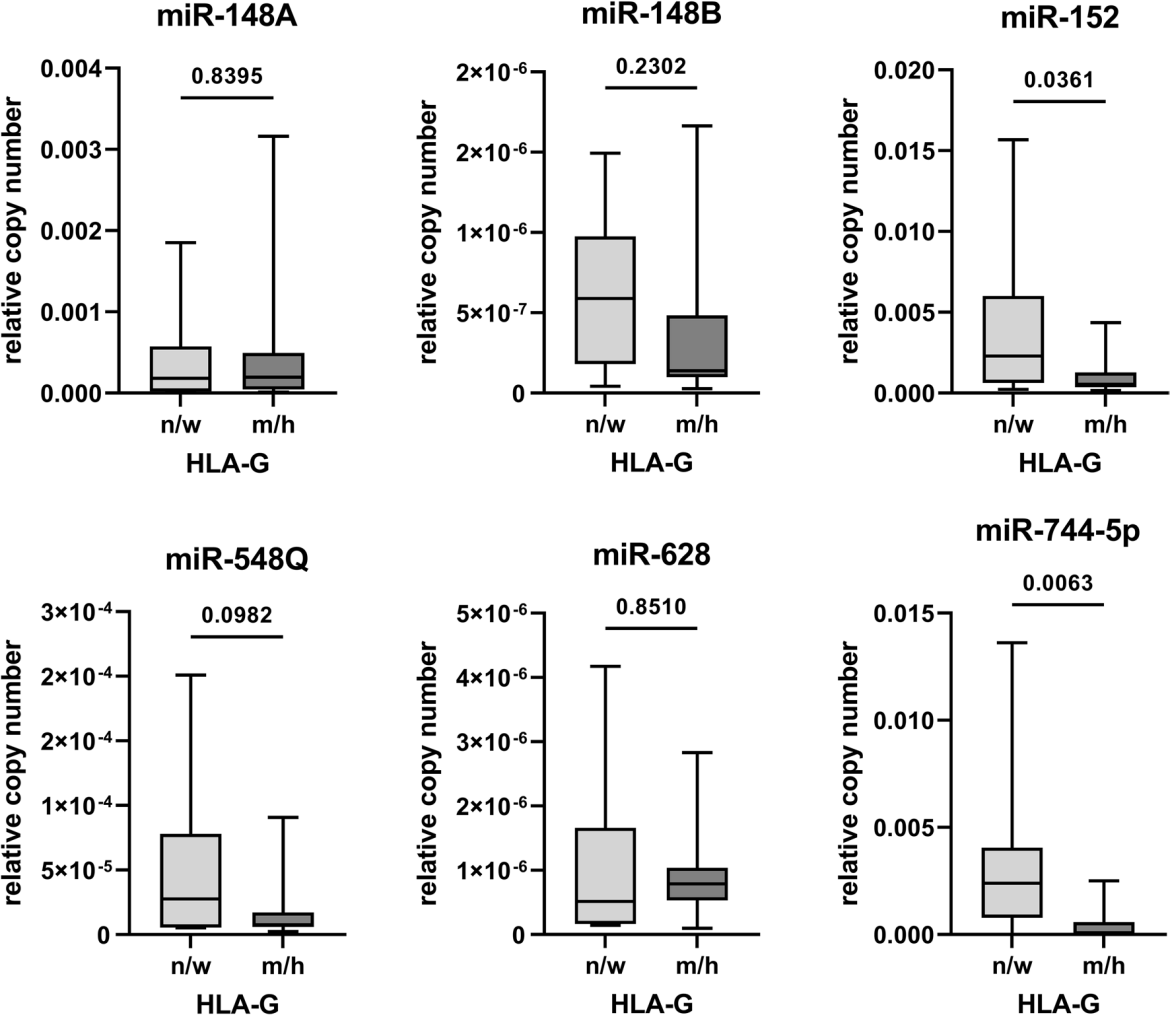
Correlation of HLA-G miRNA expression with HLA-G intensity in lung sections of SARS-CoV-2 patients. Expression of the relative copy numbers of HLA-G-regulating miRNAs was determined by qPCR on the lung sections of SARS-CoV-2 patients. The results are shown as Box-Whiskers Plots upon subdivision of the patients into HLA-G negative/weak (*n* = 8) and HLA-G medium/high (*n* = 17) based on the H-score obtained by IHC staining (HLA-G negative = 0; HLA-G weak < 50; HLA-G medium > 50 and < 100; HLA-G high > 100). The statistical significance (*p* value) was determined by calculation of the two sided student’s *t* test

Building on the miRNA-mediated posttranscriptional regulation of HLA-G expression [20–23, 68], the expression of HLA-G-specific miRNAs known to inhibit HLA-G expression by either binding to the 3’ UTR or CDS was analyzed by qPCR in lung tissues across the samples and correlated with the HLA-G staining intensity (H-score) obtained by IHC (Fig. 1b). The HLA-G protein levels of FFPE specimen were categorized into a HLA-G negative/weak (*n* = 8; HLA-G negative with H-score = 0; HLA-G weak H-score < 50) and a HLA-G medium/high (*n* = 17; HLA-G medium H-score > 50 and < 100; HLA-G high H-score > 100) group. A statistically significant, inverse expression of HLA-G-regulating miRNAs and HLA-G protein was detected for miR-744-5p (*p* = 0.0063) and for miR-152 (*p* = 0.036), which bind to the CDS and the 3ʹ UTR of the HLA-G mRNA, respectively, whereas the expression of miR-548q (*p* = 0.0982) and miR-148B (*p* = 0.2302) did not reach statistical significance (Fig. 2). The miRNAs miR-628 and miR-148A completely lacked an inverse expression to the HLA-G protein (Fig. 2). It is noteworthy that miR-744-5p and miR-152 classified as key regulators of HLA-G in previous studies [23] displayed the highest abundancies, strengthening their importance in the post-transcriptional regulation of HLA-G in tissues of SARS-CoV-2-diseased individuals.

### Effect of SARS-CoV-2-infection on immune cell infiltration in lung tissues and correlation with HLA-G expression levels

**Fig. 3:**
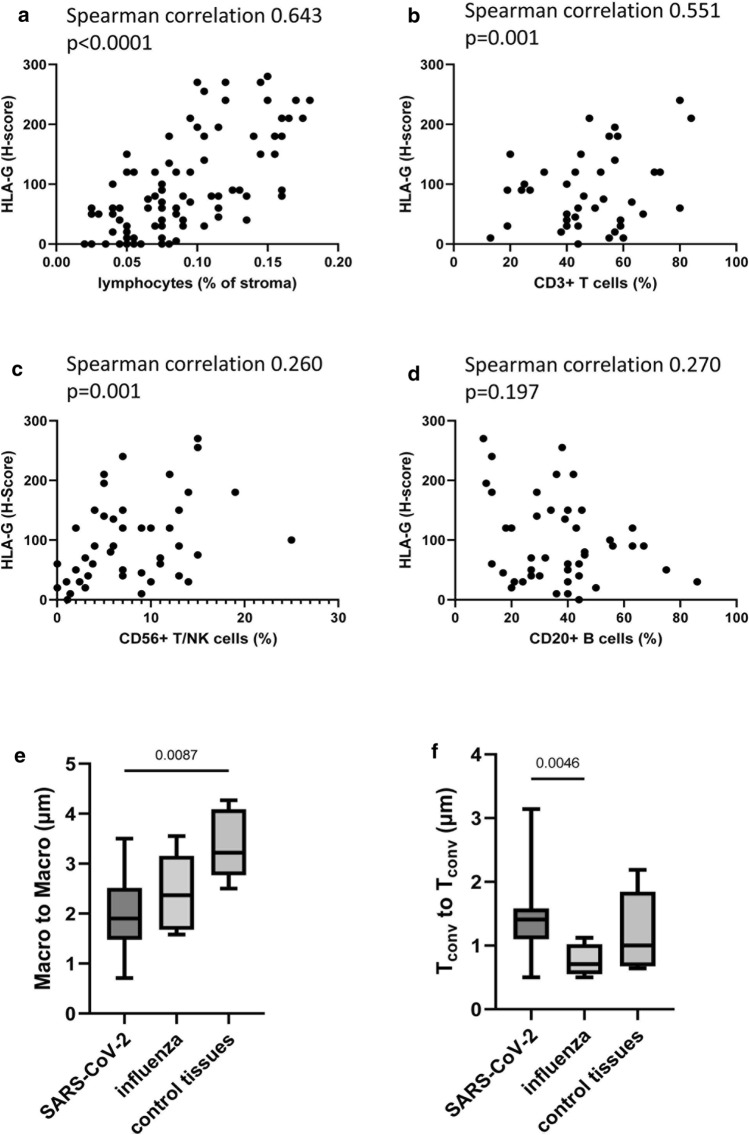
Altered composition of the immune cell infiltrate in lung tissues of SARS-CoV-2-infected patients. Lung tissues from the SARS-CoV-2 patients (*n* = 65) were evaluated for lymphocyte infiltration by HE staining (**a**) or by conventional IHC using mAbs directed against CD3 for total T cells (**b**), CD56 for NK or NKT cells (**c**) and CD20 for B cells (**d**). The resulting cell frequencies are shown in correlation to the H-score of HLA-G. In addition, the Spearman correlation coefficients and p values are given. Lung tissues from SARS-COV-2-diseased patients were stained by MSI and evaluated as described in "Material and Methods". The minimum distance (in μm) between macrophages and macrophages (**e**) as well as between conventional T cells (*T*_conv_, i.e. CD3^+^ Foxp3^neg^) to T_conv_ cells (**f**) is shown as Box-Whiskers plot. The lines represent the median values of the groups

To investigate whether there exists a link between HLA-G expression levels and the cellular environment as well as its spatial organization, the immune cell infiltration of tissues across all samples was assessed by quantification of lymphocyte infiltration using HE staining, conventional IHC and MSI (Fig. 3). An increased immune cell infiltration in the lungs of SARS-CoV-2- and to a lesser extent of influenza-infected patients compared to healthy controls was observed (data not shown). Lymphocyte quantification in tissues from SARS-CoV-2-infected patients demonstrated a statistically significant positive correlation of lung-specific HLA-G expression levels and the immune cell density (Fig. 3a). Furthermore, in these patients, pulmonary HLA-G expression was positively correlated with the frequency of CD3^+^ T cells and CD56^+^ T/NK cells analyzed by conventional IHC (Fig. 3b, c). In contrast, there was no correlation between HLA-G expression and the frequency of CD20^+^ B cells (Fig. 3d). Interestingly, lung tissues with detectable SARS-CoV-2 nucleocapsid antigen showed a slightly lower lymphocyte infiltration (data not shown).

The analysis of the frequency and composition of the immune cell repertoire was extended by a more detailed examination of the spatial distribution between the different immune cells (Fig. 3e, f). The spotwise evaluation of MSI demonstrated no significant differences in the number of infiltrating immune cells, such as CD163^+^ macrophages or total CD3^+^ T cells across the samples (data not shown). However, evaluation of the relative distance among the different immune cells in the lung epithelia highlighted that in the SARS-CoV-2 patient group the macrophages were in closer proximity to each other, while the conventional T cells determined as CD3^+^ Foxp3^neg^ cells were more distant from each other than in the tissues of controls or influenza-infected patients, respectively (Fig. 3e, f).

**Fig. 4:**
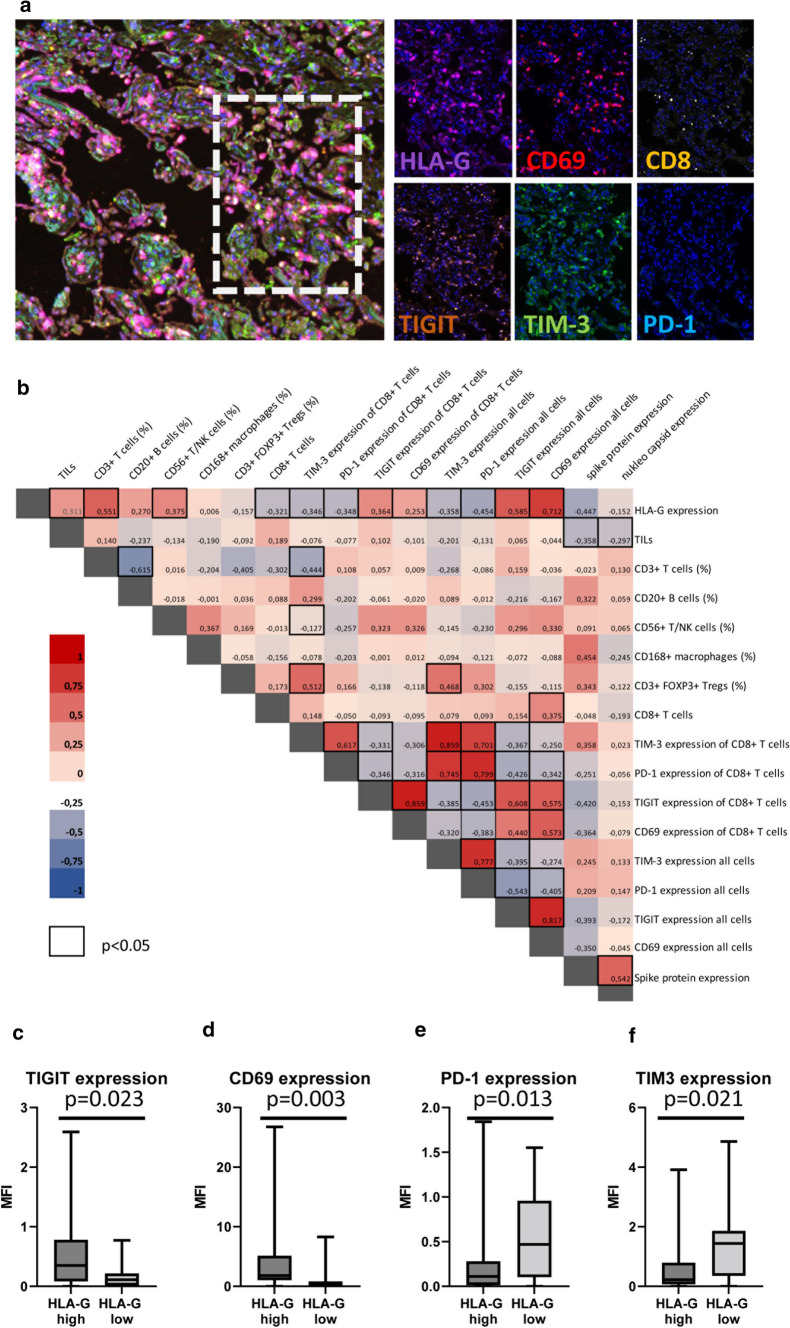
Correlation of the frequency and function of CD8^+^ T cells to HLA-G expression. MSI staining was performed as described in Materials and Methods and staining patterns of exhaustion/hyperactivation markers were correlated to HLA-G. **a** Representative multiplex staining of lung tissues from a SARS-CoV-2 patient with a multiplex panel consisting of mAbs directed against HLA-G (violet), CD69 (red), CD8 (yellow), TIGIT (orange), TIM-3 (green), and PD-1 (turquoise). **b** Pearson correlation map with association to functional markers: Pearson correlation coefficients are represented by different colors defined in the scale bar on the right side of the correlation map. Significant associations are highlighted by a black frame. Correlation of TIGIT (**c**), CD69 (**d**), PD-1 (**e**) and TIM-3 (**f**) expression to HLA-G^high^ and HLA-G^low^ expression levels

Since viral infections have been shown to influence T cell function by inducing hyperactivation and anergy [69], the frequency of CD8^+^ T cells as well as the expression of the T cell immunoreceptor with Ig and ITIM domains (TIGIT), TIM-3, programmed cell death protein 1 (PD-1), and CD69 was evaluated as surrogate markers by MSI. Data were correlated with the SARS-CoV-2-induced HLA-G expression as representatively shown in Fig. 4a. Higher HLA-G expression levels correlated with lower CD8^+^ T cell frequencies (Fig. 4b). These CD8^+^ T cells showed a significantly increased expression of CD69 and TIGIT (Fig. 4c, d), while the expression of TIM-3 and PD-1 (Fig. 4e, f) was significantly lower in high HLA-G-expressing cases. However, even though the activation marker CD69 was significantly higher expressed in these T cells, CD69^+^ T cells were more distant to high HLA-G-expressing lung epithelial cells with a minimal distance of 1.58 µm versus 3.08 µm, and an average distance of 4.73 µm versus 118.23 µm.

### Clinical relevance of HLA-G expression, frequency, and composition of the pulmonary immune cell infiltrate in SARS-CoV-2-infected patients

**Fig. 5:**
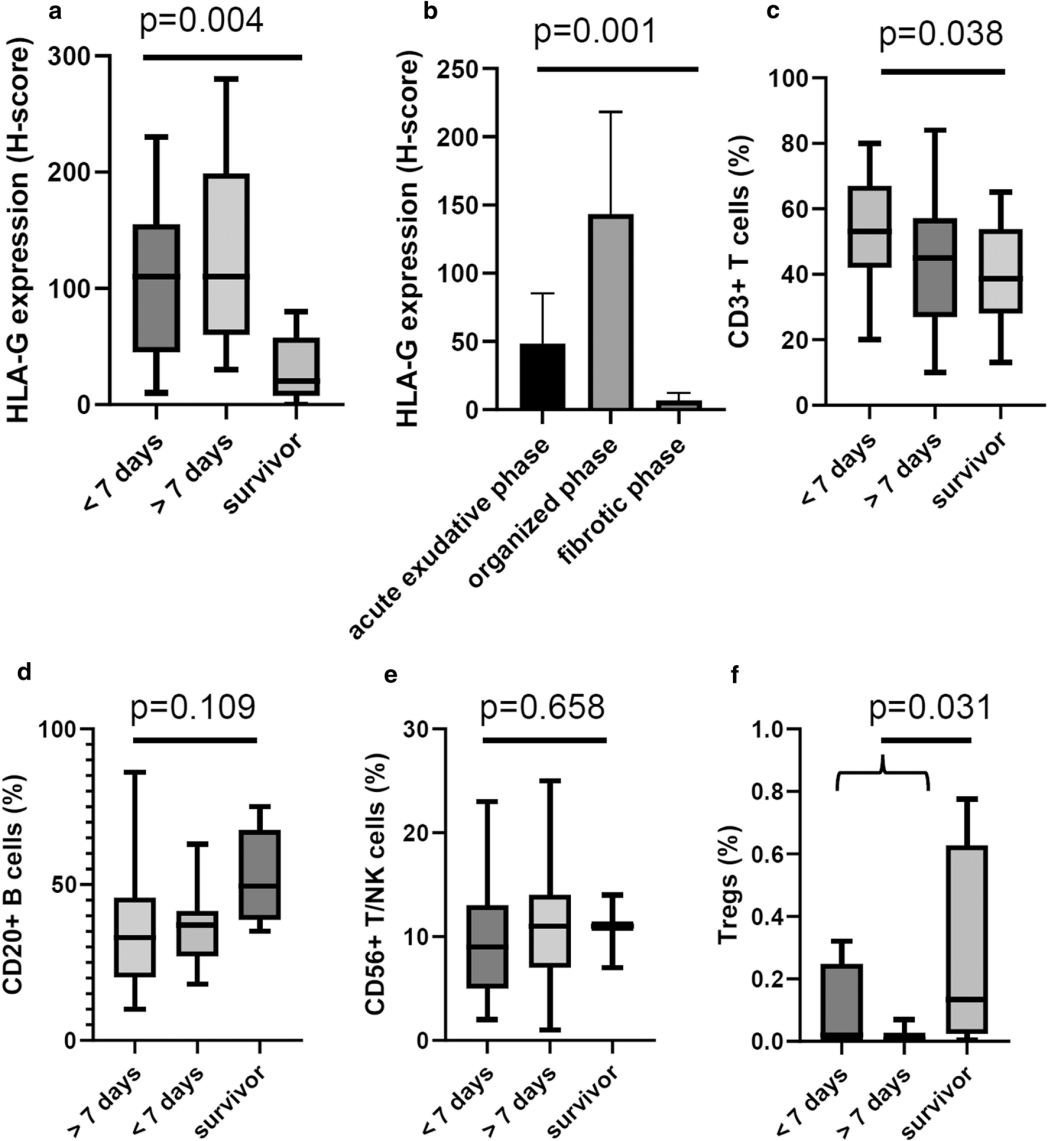
Clinical relevance of HLA-G expression and immune infiltration. HLA-G expression was correlated with the survival of SARS-CoV-2-infected patients (**a**) as well as to their histological pattern of acute interstitial pneumonia (**b**), whereas the frequencies of the different immune cell subpopulations were correlated only with survival (**c**–**f**), evaluated as the time from the first symptoms to death (< 7 days and > 7 days) or survival

To determine whether HLA-G expression levels might have a prognostic impact in SARS-CoV-2-infected lungs, the level of HLA-G expression was correlated to the severity of disease. Therefore, the patients with SARS-CoV-2 infection were divided into those who survived and those with early and late disease depending whether death occurred before or after 7 days from the start of respiratory symptoms. In SARS-CoV-2-infected patients HLA-G and nucleocapsid expression levels were associated with the patients’ outcome as shown in Fig. 5. In detail, lung resection tissues of the six SARS-CoV-2 survivors exhibited significantly lower HLA-G levels than lung tissues from deceased patients (Fig. 5a). Among the deceased individuals the HLA-G expression was lower in patients, who survived longer than 7 days compared to patients with a survival of less than 7 days after the first symptoms (Fig. 5a). Interestingly, most patients with detectable SARS-CoV-2 nucleocapsid antigen expression in lung epithelia died early (< 7 days after first symptoms, data not shown). It is noteworthy that lung samples with an early acute interstitial pneumonia pattern (exudative phase) showed lower HLA-G expression levels compared to samples with later inflammatory stages (organized and fibrotic phases) (Fig. 5b).

In addition, the correlation of the frequency and localization of lymphocytes to the survival of SARS-CoV-2-infected patients demonstrated a significantly lower frequency of CD3^+^ T cells in the stroma of lung tissues of survivors (Fig. 5c). In addition, despite not reaching statistical significance, survivors exhibited higher levels of CD20^+^ and CD56^+^ cells (Fig. 5d, e). Moreover, a significantly enhanced presence of regulatory T cells (Tregs) determined as CD3^+^ Foxp3^+^ cells could be highlighted in the SARS-CoV-2 survivors versus deceased patients (Fig. 5f) although their frequency was low.

## Discussion

Recently, HLA-G has been shown to be upregulated by various viruses, such as HCV, HCMV, HPV and HIV [32, 70, 71], which was extended in this report to SARS-CoV-2 infection. We show here for the first time that patients infected with the SARS-CoV-2 and pulmonary disease had a high frequency of membranous HLA-G expression in the lung tissues, predominantly the alveocytes, but not in other tissues suggesting an organ-specific HLA-G neo-expression. Despite sharing a viral pathogenic origin and a similar damage pattern within the lung, the level of HLA-G expression in lung epithelia of COVID-19 patients was higher than that of influenza-diseased patients and might reflect an altered immune response associated with an increased fibrosis score in the lungs of COVID-19 patients [72, 73]. In contrast, lung epithelia of non-virus-infected controls lacked HLA-G expression.

There exist a number of publications on the immune cell repertoire and/or its spatial distribution in COVID-19 damaged lungs ranging from macroscopic to single-cell level [74], but a correlation between HLA-G expression, the immune cell composition, and the clinical course has not yet been described. In agreement with published data using targeted spatial transcriptomics, changes in the cell type composition and interactions between macrophages and T cells using IHC and MSI were found in the diseased lungs. This study showed for the first time that the increased immune cell infiltration was associated with high HLA-G-expression levels, which might be due to cytokines secreted by T cells and macrophages, like IL-10, TGF-β and/or IFN-γ, present in high numbers of the lung tissue microenvironment [75, 76]. Furthermore, higher HLA-G expression levels were associated with decreased frequencies of CD69^+^ CD8^+^ T cells. CD8^+^ T cells expressing the activation marker CD69 were more distant located to HLA-G^high^ cells compared to cases with low or no HLA-G expression. In this context, it is noteworthy that HLA-G inhibits T cell activity by binding to the ILT2 and ILT4 receptors, and therefore activated CD8^+^ T cells in the proximity of HLA-G expressing cells are not functional [77, 78]. The expression of HLA-G and its receptors (ILT-2, ILT-4, KIR-2DL-4) could be also found in peripheral immune cells, like T and B cells as well as monocytes upon SARS-CoV-2 infection [59]. CD8^+^ T cells have been shown to overexpress CD69 and TIM-3 in the peripheral blood of COVID-19-infected patients compared to healthy controls, which is a characteristic for a hyperactivated/exhausted T cell phenotype. This observation has also clinical relevance, since the hyperactivated T cell status was accompanied by a reduced survival of COVID-19-infected patients [54]. Based on the analysis of PBMC in COVID-19-infected patients, CD8^+^ T cells exhibit an exhausted phenotype characterized by the surface expression of TIGIT, TIM-3 and/or PD-1 [79, 80]. In our study, TIGIT expression was significantly increased in HLA-G-positive cases, while PD-1 and TIM-3 exhibited a significantly higher surface expression on CD8^+^ T cells, which reflect an exhausted CD8^+^ T cell phenotype and mainly confirmed the published results of PBMC analysis of COVID-19-infected individuals. Dysfunction or T cell exhaustion result in deficient T cell responses in COVID-19-infected individuals. This might be driven by a type I IFN-induced transcriptional network regulating the expression of co-inhibitory molecules [81].

In addition, a negative correlation between the expression of HLA-G, the nucleocapsid, and the spike protein was detected, which point to an altered expression of these molecules during the disease and indicate an up-regulation of viral proteins in the early phase of COVID-19. The negative correlation between HLA-G expression and COVID-19 course underlines that HLA-G might be a potential therapeutic target in this disease. This hypothesis is further supported by an association between the HLA-G variant rs9380142 and the susceptibility to SARS-CoV-2 infection [82].

The molecular mechanisms regulating HLA-G expression are highly complex and comprise single nucleotide polymorphisms (SNPs) in the CDS and 3ʹ UTR of the HLA-G gene, epigenetic, transcriptional as well as posttranscriptional regulation. In multiple tumor entities, the expression of HLA-G-regulating miRNAs has been shown to be inversely correlated with HLA-G [21, 23]. In this study, we observed that lung biopsies from COVID-19-diseased patients displaying increased HLA-G protein expression exhibit lower miR-744-5p and miR-152 expression levels suggesting a deregulated HLA-G-specific miRNA expression due to COVID-19 infection. However, further evaluations are required to identify the processes responsible for directly or indirectly leading to a down-regulation of these miRNAs as well as to understand why the other known HLA-G-regulating miRNAs are not inversely expressed.

Other mechanisms resulting in HLA-G neo-expression are related to the composition of the tissue microenvironment, in particular of immune-suppressive inflammatory cytokines, such as IL-10, TGF-β, and IFN-γ, which are able to upregulate and/or enhance HLA-G expression [83]. Therefore, analysis of cytokine secretion by the different immune cell subpopulations in the SARS-CoV-2-infected lungs might help to evaluate their role in HLA-G neo-expression and in the outcome of SARS-CoV-2-infected patients.

## Conclusions

We here propose that HLA-G is a major player in the altered immunogenicity of SARS-CoV-2-infected lung epithelia. Thus, HLA-G might serve as prognostic marker and might also pave the way to develop HLA-G as therapeutic target for the treatment of COVID-19 infection by restoring the exhausted immune responses induced by HLA-G.

## Data Availability

The datasets generated during and/or analyzed during the current study are not publicly available, since no repository exists, but are available from the corresponding authors.

## Abbreviations

Ab: Antibody
ACE2: Angiotensin-converting enzyme 2
ARDS: Acute respiratory distress syndrome
CDS: Coding sequence
CoV-2: Coronavirus-2
COVID-19: Coronavirus disease-2019
CTL: Cytotoxic T lymphocytes
DFS: Disease-free survival
ELISA: Enzyme-linked immunosorbent assay
FFPE: Formalin-fixed, paraffin-embedded
G-CSF: Granulocyte colony stimulating factor
GM-CSF: Granulocyte–macrophage colony stimulating factor
HLA: Human leukocyte antigen
HLA-G: Human leukocyte antigen-G
HE: Hematoxylin and eosin
HOTAIR: HOX transcript anti-sense RNA
IDO: Indoleamine 2,3-dioxygenase
IFN: Interferon
IHC: Immunohistochemistry
IL: Interleukin
mAb: Monoclonal antibody
miRNA: microRNA
MMP: Matrix metalloproteinase
MSI: Multispectral imaging
NK: Natural killer
OS: Overall survival
PD-1: Programmed cell death protein 1
PD-L1: Programmed death ligand 1
RBP: RNA-binding protein
ROI: Region of interest
RT-PCR: Real-time polymerase chain reaction
SARS-CoV-2: Severe acute respiratory syndrome coronavirus-2
sHLA-G1: Soluble HLA-G1
TGF: Transforming growth factor
TIGIT: T cell immunoreceptor with Ig and ITIM domains
TIL: Tissue-infiltrating lymphocyte
TIM-3: T cell immunoglobulin and mucin domain 3
TNF: Tumor necrosis factor
UTR: Untranslated region
WHO: World Health Organization
